# Small Molecule Attenuates Bacterial Virulence by Targeting Conserved Response Regulator

**DOI:** 10.1128/mbio.00137-23

**Published:** 2023-04-19

**Authors:** Chang Liu, Hua Zhang, Xian Peng, Meghan S. Blackledge, Robert E. Furlani, Haoting Li, Zhaoming Su, Roberta J. Melander, Christian Melander, Suzanne Michalek, Hui Wu

**Affiliations:** a Department of Pediatric Dentistry, University of Alabama at Birmingham Schools of Dentistry and Medicine, Birmingham, Alabama, USA; b Department of Integrative Biomedical & Diagnostic Sciences, Oregon Health & Science University School of Dentistry, Portland, Oregon, USA; c Department of Chemistry and Biochemistry, University of Notre Dame, Notre Dame, Indiana, USA; d Department of Microbiology, University of Alabama at Birmingham Schools of Dentistry and Medicine, Birmingham, Alabama, USA; Columbia University

**Keywords:** biofilms, response regulator, small molecule, virulence

## Abstract

Antibiotic tolerance within a biofilm community presents a serious public health challenge. Here, we report the identification of a 2-aminoimidazole derivative that inhibits biofilm formation by two pathogenic Gram-positive bacteria, Streptococcus mutans and Staphylococcus aureus. In S. mutans, the compound binds to VicR, a key response regulator, at the N-terminal receiver domain, and concurrently inhibits expression of *vicR* and VicR-regulated genes, including the genes that encode the key biofilm matrix producing enzymes, Gtfs. The compound inhibits S. aureus biofilm formation via binding to a Staphylococcal VicR homolog. In addition, the inhibitor effectively attenuates S. mutans virulence in a rat model of dental caries. As the compound targets bacterial biofilms and virulence through a conserved transcriptional factor, it represents a promising new class of anti-infective agents that can be explored to prevent or treat a host of bacterial infections.

## INTRODUCTION

Biofilms are ubiquitous in nature and mediate a large number of infectious diseases (1, 2). Microbes residing within a biofilm exhibit high levels of tolerance to traditional antibiotics as a result of slow metabolic rates and the presence of a complex extracellular matrix that provides a physical barrier. The biofilm matrix consists of diverse biomolecules, such as polysaccharides, lipids, proteins, and nucleic acids (2, 3). Given the robust antimicrobial tolerance presented by biofilms, numerous efforts have been made to develop therapeutic strategies that circumvent this antibiotic tolerance. A variety of small molecules that inhibit biofilm formation have been identified (4), including several 2-aminoimidazole (2-AI) derivatives that are based upon the marine alkaloids oroidin and bromoageliferin. The 2-AI compounds inhibit and disperse biofilms across a broad spectrum of bacteria (5, 6), including Streptococcus mutans (7) and Staphylococcus aureus (8).

S. mutans plays an important role in the initiation and progression of dental caries and possesses several key virulence traits as follows: adhesion, production of a biofilm matrix, biofilm development and maturation, acidogenicity, and acid tolerance (9). Genes that regulate S. mutans virulence have been investigated extensively (10–12), and emerging studies have revealed a prominent role played by two-component regulatory systems (TCSs) in modulating bacterial fitness and virulence (13). A typical TCS consists of a sensor histidine kinase and a response regulator, where the histidine kinase senses an environmental signal and relays the signal to a cognate response regulator, which in turn modulates expression of a cascade of effector genes. TCSs have been previously investigated as drug targets (14, 15). Most efforts to date have been focused on targeting the histidine kinase family (16, 17); however, the response regulator is also an attractive target (18, 19).

S. mutans possesses 14 TCSs (20), including VicRK, homologues of which have been characterized in other streptococci and staphylococci (21–27). VicRK mediates the expression of genes involved in sucrose metabolism and biofilm formation in S. mutans (24). VicR and its homologs are essential not only in S. mutans (21) but also in Streptococcus pneumoniae and S. aureus (28–31).

S. aureus, a versatile pathogen, causes a wide range of illnesses, from mild skin infections to life-threatening disease (2, 32, 33). Increased antibiotic resistance by S. aureus presents a serious public health challenge, and new therapeutic agents effective against S. aureus are urgently needed. In S. aureus, a VicR homolog mediates autolysis and biofilm formation (28–30). Thus, VicR-type response regulators are attractive targets for the development of antibacterial agents (18, 19).

In this study, we report the identification of a new 2-AI derivative that selectively inhibits pathogenic biofilms through targeting response regulator VicR. A library of 2-AI derivatives was screened for antibiofilm activity against S. mutans and S. aureus, and a potent 2-AI compound was identified. Using a pulldown assay with a biotinylated analog of the active 2-AI, we determined that VicR and its homolog are the target of this class of small molecules in both S. mutans and S. aureus. Furthermore, the 2-AI attenuates S. mutans virulence *in vivo*. The lead compound is a promising anticaries agent and may have broad implications in the development of effective therapeutics toward treating other biofilm-mediated infections.

## RESULTS

### Effects of 2-AI derivatives on biofilm formation by S. mutans and S. aureus.

**FIG 1:**
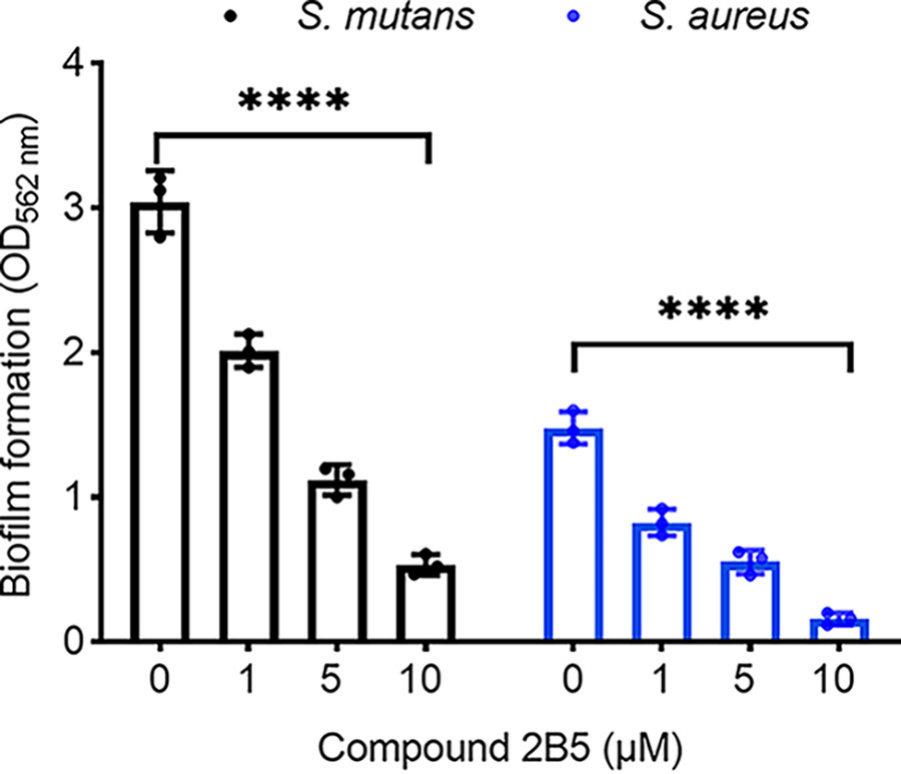
AI derivative, 2B5 inhibited S. mutans, and S. aureus biofilms. S. mutans and S. aureus biofilms treated with or without 2B5 at various concentrations and examined by crystal violet staining. ***, *P < *0.001 (ANOVA test).

The marine alkaloids oroidin and bromoageliferin were originally isolated from marine sponges and shown to possess antibiofilm activity (34, 35). Using these alkaloids as a scaffold, a synthetic library of 2-AI derivatives and related analogues of ~1,500 compounds was constructed as described (5, 6). We screened this library for compounds that selectively inhibited biofilm formation by S. mutans and identified a compound designated as 2B6 (Table 1), a member of the reverse amide class of 2-AI compounds (6). The compound 2B6 potently inhibited biofilm formation by S. mutans with an IC_50_ (concentration at which biofilm formation is reduced by 50% compared to that of the untreated control) of 14 μM but did not inhibit biofilm formation by the commensal oral streptococci, Streptococcus gordonii and Streptococcus sanguinis at concentrations up to 100 μM (data not shown). To determine the structure activity relationship (SAR) of this scaffold, we analyzed the activity of a series of analogues with varying alkyl chain lengths (compounds 2B4, 2B5, 2B7, 2B10, and 2C2) (Table 1). As the chain length increases, the IC_50_ of the derivatives decreases accordingly. Compound 2B5, possessing a 16-carbon alkyl chain, was the most potent small molecule against S. mutans (IC_50_ of 1.9 μM) (Table 1 and Fig. 1).

**TABLE 1:** Structure and activity relationship studies of inhibitors of S. mutans biofilms

Compounds (n)	IC_50_ (μM)	SD	Structure
2B6 (10)	14.063	0.351	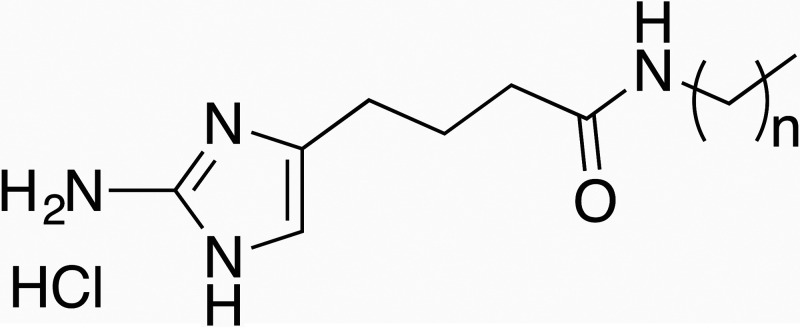
2B10 (11)	6.912	0.147	
2C2 (12)	3.991	0.049	
2B7 (13)	3.423	0.158	
2B5 (15)	1.910	0.027	
2B4 (17)	2.832	0.098	

We have previously determined that other 2-AI containing compounds inhibit biofilm formation by S. aureus (5, 36), so we also evaluated effects of 2B5 on S. aureus. As anticipated, 2B5 exhibited potent antibiofilm activity against S. aureus as well, exhibiting an IC_50_ of 3.5 μM (Fig. 1).

### The lead 2-AI compound, 2B5, targets VicR, a key response regulator in both S. mutans and S. aureus.

**FIG 2:**
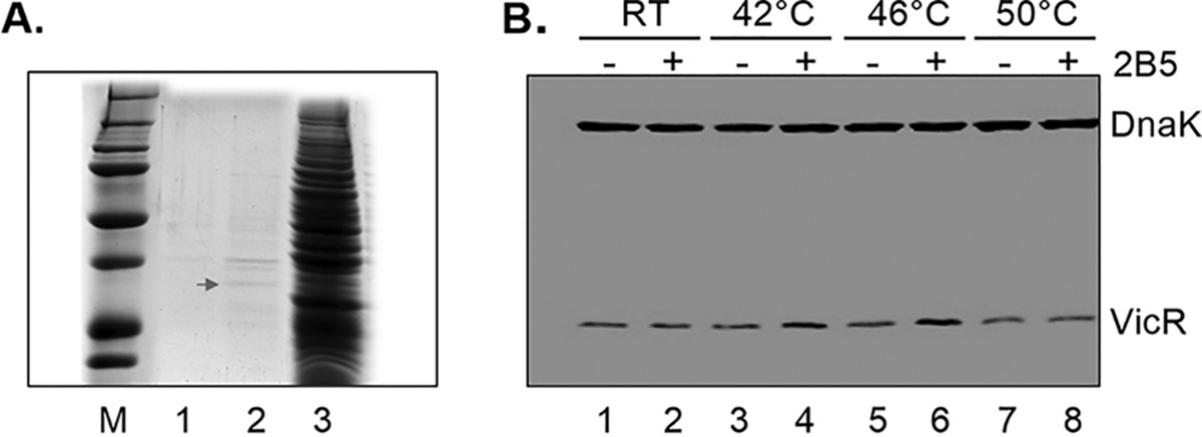
2B5 bound to VicR, a response regulator in S. mutans. (A) Purification and identification of 2-AI binding protein(s) from S. mutans. S. mutans cell lysates were incubated with biotin (lane 1) or biotinylated 2-AI (lane 2), subjected to the purification procedure with streptavidin beads, and stained by Coomassie brilliant blue. S. mutans cell lysates bound to streptavidin beads (lane 3, input control) were also stained. A major protein band presented by biotinylated 2-AI pulldown (lane 3) but absent in the biotin control (lane 2) was subjected to LC/MS/MS analysis. M represents protein markers. (B) Thermal stability of VicR determined by cellular thermal shift assay. S. mutans cell lysates treated with 2B5 and the vehicle control, 1% DMSO, were incubated at different temperatures followed by cooling down at room temperature. The heated cell lysates were subjected to centrifugation and separated into soluble fractions in supernatants and precipitates, and the soluble fractions were analyzed by SDS-PAGE followed by Western blotting analysis using a VicR- or DnaK-specific antibody.

To determine the mechanism of action by which this series of reverse amide 2-AI derivatives inhibit biofilm formation by S. mutans, we employed an affinity purification assay using a previously reported procedure (18). The biotinylated 2B5 was synthesized (see Fig. S1 in the supplemental material) and tested in the biofilm inhibition assay and found to exhibit higher activity than the parent compound (see Fig. S2 in the supplemental material). The biotinylated 2B5 was used to conduct affinity purification while 2B5 supplemented with biotin served as a control. A number of protein bands were identified as potential binding partners to the biotinylated 2-AI (Fig. 2A, lane 2), which were absent in the biotin-treated control group (Fig. 2A, lane 1). The major protein band was excised and identified as VicR by liquid chromatography with tandem mass spectrometry (LC/MS/MS) analysis. VicR is a response regulator, which plays an important role in S. mutans growth and biofilm formation. Along with its cognate histidine kinase VicK, it constitutes the VicRK TCS that is involved in regulation of cell wall metabolism, nutrient uptake, osmotic protection, and biofilm formation (25, 26, 28, 29, 37).

10.1128/mbio.00137-23.1 FIG S1 Synthesis of biotinylated 2B5. Biotinylated 2B5 was prepared as shown in Scheme S1. Briefly, hexadecylamine S1 was treated with Boc anhydride to yield S2, and then *N*-alkylated with 8-iodooct-1-yne to deliver the protected secondary amine S3. TFA-mediated removal of the Boc group generated the secondary amine S4 as the TFA salt. EDC mediated coupling between S4 and the tri-Boc protected 2-aminoimidazole carboxylic S5 acid gave amide S6, which was then subject to a copper(I)-catalyzed azide-alkyne cycloaddition with the biotin azide S7 to form triazole S8. Deprotection of the 2-AI with TFA afforded biotinylated 2B5, which was converted to a HCl salt for biological testing. Download FIG S1, PDF file, 0.3 MB. Copyright © 2023 Liu et al. 2023 Liu et al. https://creativecommons.org/licenses/by/4.0/ This content is distributed under the terms of the Creative Commons Attribution 4.0 International license.

10.1128/mbio.00137-23.2 FIG S2 Biotinylated 2B5 exhibited higher biofilm inhibitory activity than unlabeled 2B5. Biotinylated 2-AI (Bio2B5) and 2B5 precursor (p2B5) inhibited biofilms formed by S. mutans (A) and S. aureus (B). S. mutans and S. aureus were grown in 96-well plates with 2B5 and biotinylated 2B5 at different concentrations, respectively, and their biofilms were developed over a period of 16 h and measured at OD_562_ after staining with crystal violet. Download FIG S2, PDF file, 0.2 MB. Copyright © 2023 Liu et al. 2023 Liu et al. https://creativecommons.org/licenses/by/4.0/ This content is distributed under the terms of the Creative Commons Attribution 4.0 International license.

To verify binding of 2B5 to VicR, we employed a cellular thermal shift assay that has been successfully used to monitor drug binding to target proteins in cell and tissue samples (38). After heating S. mutans cell lysates to higher temperatures (42 and 46 °C), higher levels of VicR were observed in soluble fractions of 2B5-treated samples compared to untreated controls (Fig. 2B). The induction of thermal stability of VicR in the presence of 2B5 further indicated that 2B5 binds VicR.

**FIG 3:**
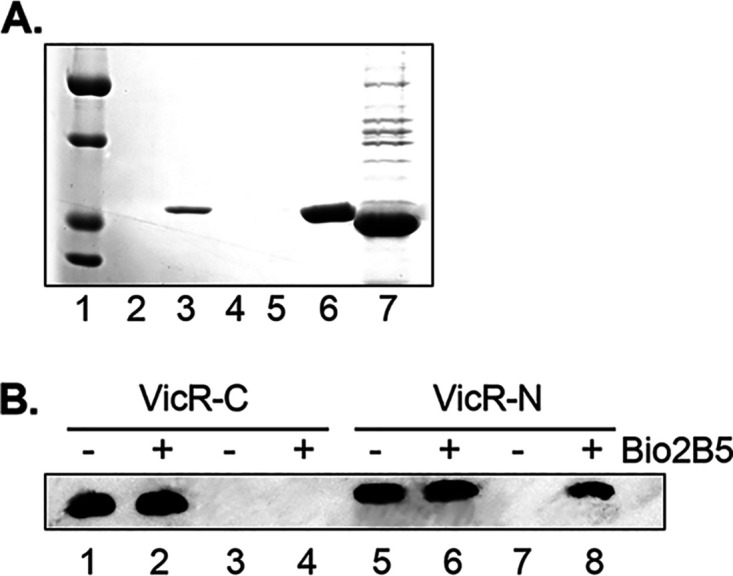
2-AI bound selectively to VicR N-terminal domain. (A) 2-AI selectively bound to recombinant VicR but not Smu.1547. Biotin and biotinylated compound were incubated with recombinant VicR and Smu_1547, respectively, and then subject to purification via streptavidin beads. Lane 1, protein marker; lanes 2 and 4, biotin-treated recombinant VicR and Smu_1547, respectively; lanes 3 and 5, biotinylated compound-treated recombinant VicR and Smu_1547, respectively; lane 6, VicR input; lane 7, Smu_1547 input. (B) 2-AI bound to N-terminal VicR (VicR-N). Recombinant 6×His tag labeled N-terminal and C-terminal VicR (VicR-C) were prepared and subjected to pulldown with streptavidin beads after incubation with either biotin or biotinylated 2-AI. VicR bound to the beads was detected by Western blotting using 6×His tag antibody.

To further determine whether active 2-AIs directly bind to VicR, we generated recombinant VicR and performed pulldown assays using the biotinylated compound. The biotinylated 2-AI (Fig. 3A, lane 3), but not the control biotin immobilized beads (Fig. 3A, lane 2), captured recombinant VicR, demonstrating binding of biotinylated 2-AI to VicR. To determine whether the binding is selective, another response regulator Smu.1547, which belongs to the NarL/FixJ superfamily (39), was included as a control (Fig. 3A, lanes 4 and 5). The biotinylated 2-AI failed to capture recombinant Smu.1547 (Fig. 3A, lanes 4 and 5), indicating its selective binding to VicR.

VicR possesses two domains, an N-terminal signal receiver domain and a C-terminal DNA binding domain. To determine to which domain the 2-AI binds, recombinant N-terminal VicR (VicR-N) and C-terminal VicR (VicR-C) were generated and examined in the pulldown assay with the biotinylated 2-AI. Western blotting revealed that VicR-N and not VicR-C was captured by the biotinylated 2-AI (Fig. 3B, lanes 4 and 8), suggesting that the N terminus binds to this reverse amide 2-AI.

**FIG 4:**
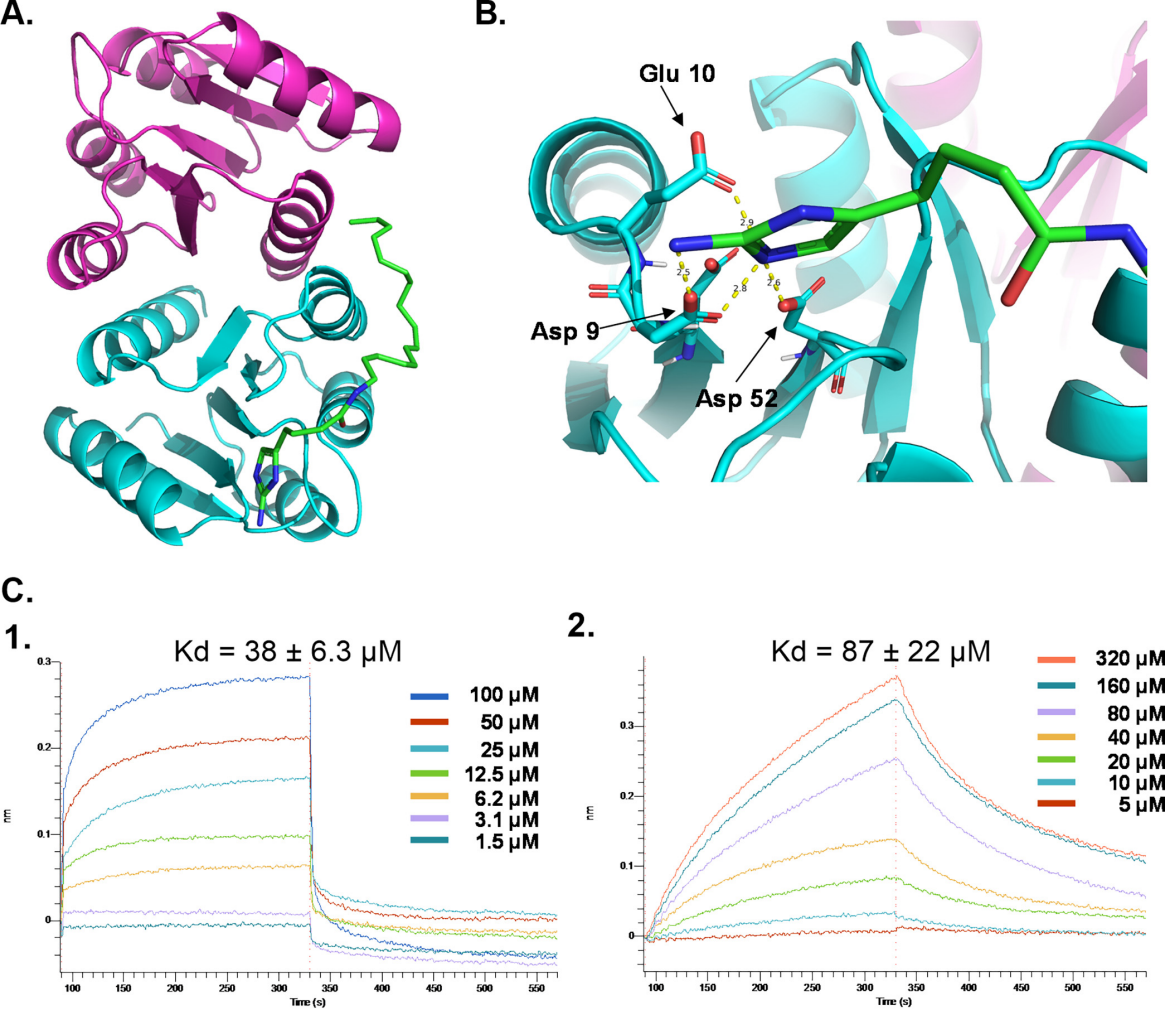
Structural and biophysical studies of VicR and 2B5. The crystal structure of VicR-N was determined. (A) Ribbon representation showing two molecules of VicR-N (cyan and purple) in the complex with 2B5 (green). 2B5 was docked to the VicR-N structure determined experimentally. (B) Unmodeled density from the structural map of VicR-N and the distance between 2B5 and the amino acids surrounded the density. (C) Binding kinetic analysis of 2B5 and VicR. Real-time association and dissociation analysis of VicR to biotinylated 2B5 (1) or to unlabeled 2B5 (2).

To define the binding of VicR-N to 2B5, we attempted to solve the crystal structure of VicR-N in the presence of 2B5. We obtained diffractable crystals and determined the crystal structure of VicR-N using molecular replacement. VicR-N forms a dimer in the asymmetric unit (Fig. 4A). We also identified an unmodelled density surrounded by four negatively charged amino acids (Asp8, Asp9, Asp52, and Glu10) within the experimental structural map (Fig. 4B). To further investigate the binding, we docked 2B5 to VicR-N using AUTODOCK 4 and determined a binding energy of −4.98 kcal/mol. In the docking model, 2B5 is close to Asp9 (2.5 Å), Asp52 (2.6 Å), and Glu 10 (2.9 Å) of VicR-N, three of the negatively charged amino acids surrounding the undefined density shown in the experimental structure map (see Fig. S3 in the supplemental material).

10.1128/mbio.00137-23.3 FIG S3 The binding pocket identified from the structural map of VicR-N. (A) A density exists in the experimental structure map that is surrounded by four negatively charged amino acids. (B) The four negative charged amino acids were labeled with arrows. Download FIG S3, PDF file, 0.5 MB. Copyright © 2023 Liu et al. 2023 Liu et al. https://creativecommons.org/licenses/by/4.0/ This content is distributed under the terms of the Creative Commons Attribution 4.0 International license.

Furthermore, we performed bio-layer interferometry studies using the Octet Red system to determine interactions between VicR and 2B5. The binding kinetic parameter was calculated by a series of titration of 2B5 and fitting based on global 1:1 analysis. These analyses determined that the dissociation rate (*K_d_*) value for the binding of 2B5 to VicR is at 87 ± 22 μM (Fig. 4C). Given the high activity in the inhibition of S. mutans biofilms exhibited by the biotinylated 2B5 we also determined its binding *K_d_* to be at 38 ± 6.3 μM (Fig. 4C). The high binding affinity from the biotinylated 2B5 may explain why it exhibited higher inhibitory activity against S. mutans biofilms. Together, these data demonstrated the binding of 2B5 to VicR and illustrated how 2B5 inhibited VicR-mediated biofilm formation.

**FIG 5:**
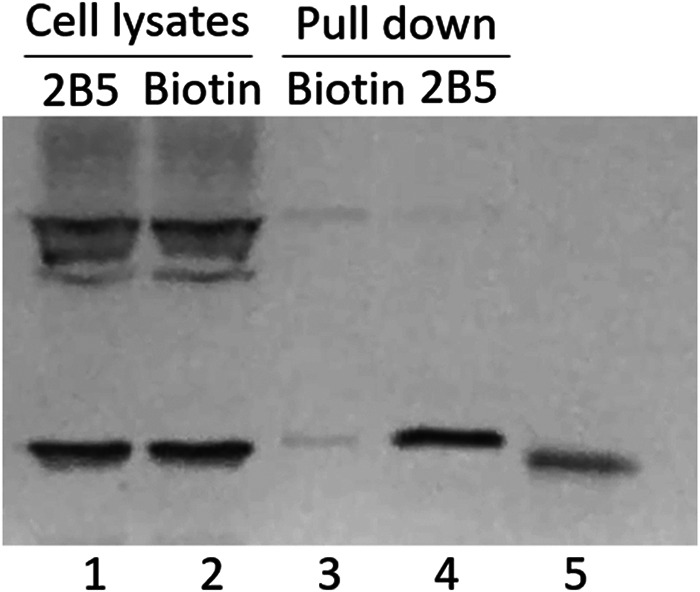
Biotinylated 2-AI bound to a VicR homolog in S. aureus. S. aureus cell lysates were incubated with either biotin conjugated 2-AI (lane 1) or biotin immobilized streptavidin beads (lane 2), and bound fractions eluted from biotin (lane 3) or biotinylated 2B5 (Lane 4) pulldowns were subjected to Western blotting using VicR-specific antibody. Recombinant VicR of S. aureus was used as a positive control (lane 5).

As 2B5 also inhibits biofilm formation by S. aureus, and S. aureus possesses a VicR-like response regulator (VicR-Sa) that shares 65% identity with VicR of S. mutans UA159, we evaluated whether the biotinylated 2-AI also interacts with VicR-Sa. Cell lysates of S. aureus were incubated either with biotinylated 2-AI immobilized streptavidin beads or biotin immobilized streptavidin beads. The biotinylated 2-AI immobilized streptavidin beads captured VicR-Sa as determined by Western blotting (Fig. 5, lane 4), while the biotin immobilized streptavidin beads did not (Fig. 5, lane 3), suggesting that the reverse amide 2-AI also binds to S. aureus VicR. Interestingly, there was an additional band that reacted with the VicR-specific antibody in cell lysates of S. aureus (Fig. 5, lanes 1 and 2); however, this protein band was not captured by the 2-AI immobilized beads, further indicating the selectivity of 2-AIs toward VicR-Sa.

### Compound 2B5 inhibits expression of *vicR*.

**FIG 6:**
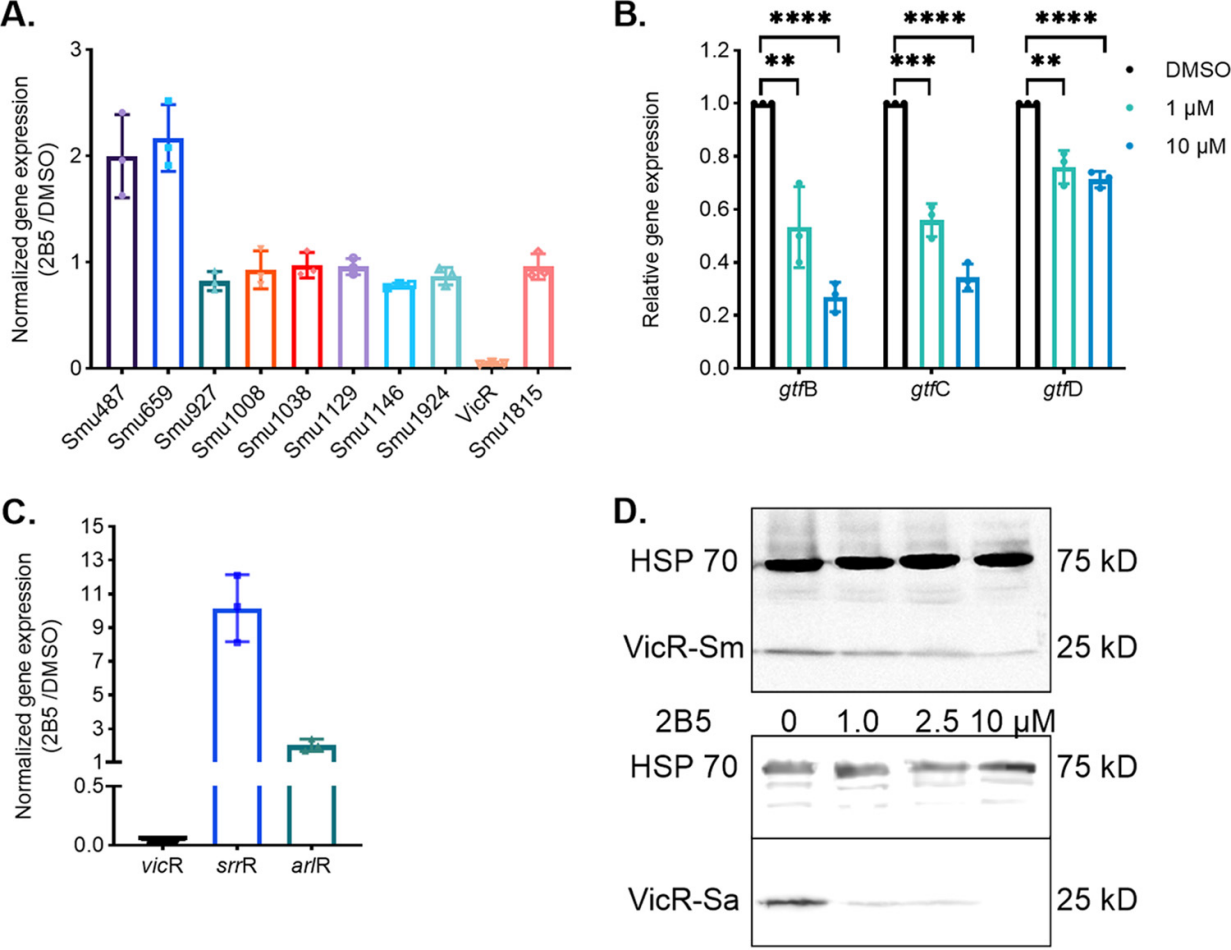
2B5 inhibited expression of *vicR* and VicR-regulated genes. (A) Effects on expression of genes encoding *vicR* and other response regulators in S. mutans. (B) Effects of 2B5 on expression of genes coding for glucosyltransferases GtfBCD in S. mutans. S. mutans cells were treated with or without 2B5, and expression of genes coding for VicR and other response regulators and GtfBCD were determined by real-time quantitative PCR using specific primer pairs (Table 3). **, *P* < 0.01; ***, *P *< 0.001; ****, *P *< 0.0001 (*t* test). (C) Effects of 2B5 on expression of genes coding for VicR and other response regulators in S. aureus. S. aureus cells were treated with or without 2B5, and expression of VicR and other response regulators was determined by real-time quantitative PCR using specific primer pairs (Table 3). Expression levels of each gene were normalized to the untreated control. (D) Effects of 2B5 on VicR protein levels by Western blotting. S. mutans and S. aureus cells were treated with or without 2B5, respectively, and cell lysates were prepared and subject to Western blotting using VicR- or HSP70-specific antibody.

As 2B5 binds to VicR and inhibits biofilm formation by S. mutans, we next determined whether 2B5 affects expression of the gene encoding VicR (*vicR*), as well as those encoding other response regulators in S. mutans by real-time reverse transcriptase PCR (RT-PCR). The expression of *vicR* was significantly downregulated by 2B5 (about 20-fold), while the expression of other response regulators was either increased (about 2-fold) or only modestly attenuated (less than 2-fold) in comparison to the vehicle control (Fig. 6A).

It is well known that VicR regulates the expression of genes encoding glucosyltransferases (Gtfs) that are key to the production of the S. mutans biofilm matrix and, therefore, important for biofilm formation (40). As 2B5 inhibits expression of *vicR* and reduces biofilm formation, we examined the effect of 2B5 on the expression of *gtf*s. 2B5 significantly inhibited expression of *gtfB* and *gtfC* and also affected expression of *gtfD* to a lesser extent (Fig. 6B). These data revealed a mechanistic connection between downregulation of *vicR* and inhibition of S. mutans biofilms by 2B5.

Similarly, we examined the effect of 2B5 on the expression of genes encoding VicR-like regulators and other response regulators in S. aureus by real-time RT-PCR. Expression of *vicR* in S. aureus was also decreased significantly (by 50-fold) upon treatment with 2B5 in comparison to the vehicle control. Expression of *srrR* and *arlR* (41), two closely related homologs, was not inhibited but increased by 2-fold (Fig. 6C). These data suggest that 2B5 also selectively inhibits expression of *vicR* of S. aureus.

To further verify the effects of 2B5 on VicR, we performed Western blotting using a VicR-specific antibody. In both S. mutans and S. aureus, VicR levels decreased in a dose-dependent manner as bacteria were treated with increased amounts of 2B5. Heat shock protein 70 (HSP70) (42) was used as a control (Fig. 6D). These results further demonstrate that the reverse amide 2B5 selectively represses the VicR regulatory system in both S. mutans and S. aureus.

### The 2AI-derivative inhibits S. mutans-induced virulence in a rat model of dental caries.

The effect of the lead 2-AI derivative 2B5 on S. mutans colonization and cariogenicity *in vivo* was evaluated in a rat model of dental caries (12). All rats from the experimental groups were colonized by S. mutans. Interestingly treatment with 2B5 at 100 μM did not significantly affect bacterial colonization; however, it significantly attenuated cariogenicity when compared to the untreated control group (Table 2). The buccal (Table 2), sulcal (data not shown), and proximal (data not shown) surface caries scores of the treated rats were all reduced. Inhibition of virulence by this compound is comparable to that observed with fluoride (Table 2); however, fluoride also modestly decreased bacterial colonization. These data suggest that the 2-AI derivative is effective in inhibiting bacterial virulence. Furthermore, the treated rats did not lose weight over the course of the study in comparison with the control group, suggesting that the compound is not toxic to the rats. These data indicate that VicR-targeted inhibition is promising for the development of anticaries therapy.

**TABLE 2:** Small molecule 2B5 attenuates S. mutans virulence^*a*^

Group	Wt (g)	Mean caries scores (CFU/mL [×10^6^])				
		Buccal				MS
		E	Ds	Dm	Dx	
2-AI	163 ± 15	8.5 ± 0.4	5.8 ± 0.5	3.3 ± 0.5	2.2 ± 0.7	4.5 ± 1.4
Vehicle	160 ± 14	13.4 ± 0.6	10.8 ± 0.5	7.6 ± 0.2	5.2 ± 0.4	4.7 ± 1.1
Fluoride	164 ± 12	7.2 ± 0.6	4.6 ± 0.7	2.0 ± 0.4	0.6 ± 0.4	3.3 ± 1.3

a E, enamel; Ds, dentinal slight; Dm, dentinal moderate; Dx, dentinal extensive. 2-AI versus vehicle, *P* < 0.001; fluoride versus vehicle, *P* < 0.001; bacterial colonization (CFU/mL), no significance; weights: no significance.

## DISCUSSION

The 2-AI-containing small molecules derived from marine alkaloids have been reported to exhibit antibiofilm properties against a wide range of pathogens (5, 36). Here, we have identified a series of 2-AI compounds that potently inhibit biofilm formation by S. mutans, the most recognized cariogenic bacterium in the oral cavity, as well as S. aureus, an important nosocomial pathogen. In addition, we have identified the response regulator VicR as the target of this series of compounds, delineated how the reverse amide 2-AI alters gene expression in cariogenic S. mutans and pathogenic S. aureus, and subsequently inhibited bacterial virulence. These findings offered new insights into the design and development of targeted antibacterial therapies.

Related reverse amide 2-AI analogues have been shown to inhibit biofilm formation by several species, including the Gram-negative pathogen Acinetobacter baumannii. In A. baumannii, the transcriptional regulator BfmR has been identified as the target of the reverse amide class of anti-bacterial agents (18). Small molecules that target response regulators such as ArsR and HsrA have been shown to have bactericidal activity against antibiotic-resistant strains of Helicobacter pylori, while they don’t have similar effects on Escherichia coli and Staphylococcus epidermidis (43, 44). A small molecule targeting the response regulator DosR of Mycobacterium tuberculosis has also been discovered using a whole cell, reporter-based phenotypic high-throughput screen. Interestingly the compound does not inhibit bacterial persistence but reduces bacterial ability to synthesize triacylglycerol by 50% via inhibiting DosR DNA binding dose-dependently (45). Whether those inhibitors act on biofilms awaits further investigation. Nevertheless, these studies highlight that bacterial response regulators can be explored as novel anti-infective targets for a wide range of bacterial infections, opening further opportunities for new therapeutics.

We have determined that the reverse amide 2-AI binds to a highly conserved response regulator VicR in S. mutans at the VicR N-terminal receiver domain. We also show that the compound binds to S. aureus VicR. Furthermore, the compound significantly downregulates the expression of *vicR* but not genes coding for other response regulators in both S. mutans and S. aureus, indicating its selectivity. Bacterial TCSs can regulate their own expression (41), and therefore, it is plausible that VicR would regulate itself by binding to its own promoter. Whether the binding by the compound 2B5 would impact VicR autoregulation and how it downregulates expression of *vicR* selectively among a dozen other response regulators in S. mutans awaits further investigation.

The *vicR* gene is essential in both S. mutans and S. aureus, which explains the observation that the compound inhibits bacterial growth at higher concentrations (data not shown). As we and others have reported, the VicRK TCS controls biofilm formation by regulating the expression of genes coding for key matrix-producing enzymes GtfBC in S. mutans (24, 46, 47), highlighting how the targeting of VicR by small molecules not only affects bacterial growth but also modulates biofilm formation. Mechanistic details underlying differential regulation of bacterial growth and biofilm remain to be fully elucidated.

Further, we have demonstrated that the lead reverse amide 2-AI binds to the N-terminal signal receiver domain of VicR. This binding may inhibit the formation of the active VicR homodimer, which occurs at the N-terminal domain (19). It is conceivable that the binding of the 2-AI could lead to conformational changes in VicR that downregulates expression of both *vicR* and VicR-regulated genes such as *gtfBCD* and *ftf* (24). It has been reported VicR is not regulated by VicKX in S. mutans (25); however, VicR may regulate itself, representing a previously unknown regulatory mechanism for the VicR regulon.

In S. aureus, binding to VicR may modulate expression of genes involved in bacterial autolysis, thereby affecting biofilm formation (29). Conformational switches triggered by ligand binding is well documented in other transcriptional factors. For example, a cognate autoinducer binds to the nascent Agrobacterium tumefaciens transcriptional regulator TraR that, in turn, alters its conformation and oligomerization (48, 49), leading to diverse cellular responses including biofilm formation. Such mechanisms are widespread in quorum-sensing regulation (27). It is also possible that inhibition by the small molecule may alter the phosphorylation of the VicR N-terminal receiver domain during activation (50). Additional studies are required to uncover these molecular details.

Given the conservation of VicR homologs, the selectivity of the compound is intriguing. It inhibits biofilm formation by S. mutans and S. aureus but did not inhibit biofilm formation by S. gordonii and S. sanguinis. It is worth noting that VicR of S. mutans shares 65% identity and 81% similarity to its homolog in S. aureus (51), but the identity is lower in both S. sanguinis (52) and S. gordonii *DL1* (53), which may explain the observed selectivity toward S. mutans and S. aureus. We and others have designed inhibitors that are selective against highly conserved enzymes such as DHFR (54) and Gtfs (55) in S. mutans and other microbes (51).

The 2-AI compound inhibited S. mutans biofilm formation dose dependently *in vitro*; interestingly, it did not significantly reduce bacterial colonization *in vivo* in a rat model of dental caries but did significantly reduce bacterial virulence (Table 2). It is possible that the dose used *in vivo* may only attenuate S. mutans virulence by selectively targeting VicR-mediated virulence such as acid production or stress responses, without reducing bacterial colonization. This *in vivo* efficacy is desirable in the development of new antibacterial therapeutics, where the fine-tuned selective targeting of bacterial virulence without affecting bacterial survival is advantageous in reducing the risk of selecting for antibiotic resistance, a major public health problem in the antibiotic-resistant era (56–58). The *in vivo* efficacy in the absence of any significant impact on bacterial colonization observed in this study is remarkable, as this would reduce the likelihood of imposing selective pressure to develop resistance, as many antivirulence strategies do inhibit bacterial burden as well (59). Understanding of the underlying mechanistic details of this inhibitor *in vivo* will help to design more robust therapeutic approaches that significantly reduce the risk of developing antimicrobial resistance, thus preventing and treating challenging biofilm-driven bacterial infections.

In conclusion, this study reports the identification of a reverse amide 2-AI lead that selectively targets an essential response regulator VicR in both S. mutans and S. aureus. This is the first report of direct binding of a 2-AI-derived compound to a response regulator in Gram-positive bacteria. Importantly, the compound also inhibited S. mutans virulence in an *in vivo* model. Mitigation of S. mutans virulence without reducing bacterial burden may also reduce the potential to develop unwanted anti-infective resistance. Further, the conservation of the target response regulator (23, 60) has the potential to allow the development of a broad-spectrum class of antivirulence agents. Such therapeutics would offer a new option to treat multidrug-resistant bacterial infections.

## MATERIALS AND METHODS

### Bacteria strains, culture conditions, and chemicals.

S. mutans UA159 and S. aureus NCTC 8325 (51) were used in this study. Strains were grown statically at 37°C on Todd-Hewitt broth (THB) or THB agar plates under an aerobic atmosphere with 5% CO_2_ (S. mutans) or without CO_2_ (S. aureus). A single colony of each strain was inoculated into 3 mL THB and incubated for 24 h at 37°C without agitation. Overnight bacterial cultures were then inoculated into fresh THB to allow bacteria to grow until they reached exponential growth phase—optical density at 470 nm (OD_470_) = 1. The exponentially grown bacteria were then inoculated to grow biofilms. Chemically defined medium-biofilm medium (BM) containing 1% sucrose as previously described (7, 54) was used to grow S. mutans biofilms; THB with 1% sucrose was used to produce S. aureus biofilms. Chemical compounds and biotinylated compounds were synthesized using previously described strategies (6, 18, 61) and dissolved in dimethyl sulfoxide (DMSO) at 100 mM.

### Formation and inhibition of S. mutans and S. aureus biofilms.

Effects of 2-AI compounds on bacterial biofilm formation were examined using 96-well flat-bottom polystyrene microtiter plates (Nalge Nunc International, Rochester, NY, USA) as previously described (7, 54). Briefly, bacteria grown to exponential phase were inoculated into wells of a 96-well plate at 1:100 dilution with designated concentrations of compounds and incubated for 16 h. The final concentration of DMSO in each well was 1%, which did not have any noted impact on bacterial growth. Bacterial growth was measured at 470 nm using a microplate reader (BioTek ELx, USA). Crystal violet staining was used to monitor biofilm formation as described previously (7, 46, 54, 62). Each assay was done in triplicate and individually repeated three times.

### Expression of genes coding for *vicR* and other response regulators and biofilm-associated factors in S. mutans and S. aureus determined by quantitative real-time RT- PCR.

S. mutans UA159 cells grown to exponential phase treated with either 10 μM 2B5 or DMSO control were collected by centrifugation at 6,000 × *g* at 4°C for 10 min. The total RNA was extracted using the same numbers of cells from controls and treated groups, respectively. All harvested cells were digested by *N*-acetylmuramidase (mutanolysin; Sigma-Aldrich, St. Louis, USA) at 20 μg/mL and lysozyme at 10 μg/mL at 37°C for 60 min. Lysed cells were then extracted with TRIzol (Invitrogen, Carlsbad, USA) and further digested by RNase-free DNase (Promega, Madison, USA) to remove trace amounts of DNA. The isolated RNA was reverse transcribed into cDNA using random primers (Promega, Madison, USA). cDNA samples were then quantified by real-time PCR using the iQ SYBR green supermix kit (Bio-Rad, Madison, USA). PCR primers used are listed in Table 3. The PCR cycle was set up as previously described (7, 54). A standard curve was generated for each gene and then plotted by amplification of a series of diluted cDNA samples. RNA samples without reverse transcription were used as negative controls to determine any potential contamination from genomic DNA. The expression levels of all selected genes were normalized using 16S rRNA as an internal standard.

**TABLE 3:** Primers for recombinant expression proteins and real-time PCR

Primer	Forward	Reverse
Smu.1517 (VicR)	CGGGATCCATGAAGAAAATTCTAATC ^*a*^	CCGCTCGAGTTAGTCATATGATTTCATG
Smu.927	CGGATCCATGTCTTTAACGATTTTACTAGC	CCGCTCGAGTCACCCGCCAATTCTAACTAAG
Smu.1517Nterm	CGGGATCCATGAAGAAAATTCTAATCGTT	CCGCTCGAGTGCGGATTCAATATTTTCAGT
Smu.1517Cterm	CGGGATCCGTGGCTGAGGAAAATGCTTCA	CCGCTCGAGTTAGTCATATGATTTCATGTA
Smu.1547	CGGGATCCATGAAATTACTAGTTGC TGAAGAT	CCGCTCGAGTTAGAACCAACCATTCTCA
Smu.487	TGTAGAAGTTATCGGTGAGG	CCAAGTCCATGACAACAAC
Smu.659	GTCAGTGCCAATCAGGTT	GTCCACATCAGGCATCATA
Smu.927	AAGACGAAGAGCAGTTGT	CGATAGCATCTTGTCCATTA
Smu.1008	AATTGACTCCAACAGAAACG	CTTAGTGACAACCTCTCCTT
Smu.1038	AGGTAATCCAAGCATACTCAG	TTCCAGGCAGCATAATGTC
Smu.1129	TCGGTTGTTGAAGTCTATGT	TCCTACACTGCGTAATGTC
Smu.1146	GGAATTGGAGTTGCGAATC	AAGTTGACCTGACAAGTAGG
Smu.1924	ACTGCTCGTGACTCTATTATG	CGATATAACCATCTGCTCCA
Smu.1517	GGCGTGAAGCATTAAGTAA	CCGTCTAGTTCTGGTAACA
Smu.1815	AGGCAATGAACATCTATCAC	AATCGCTTCTGTGGAGAC
Smu.1547	GTCATATAGTTGCGGATAGTTC	GCCTCCAATCAAACAATAGC
Smu.1070	ACTCTTCATCTTGACCTCTT	TCACGAAAGTATGCTAACAG
Smu.1409	GAGCGAGGATAATATCATAAGG	GCAGGTGGTTATTGTTTCAG
Smu.1964	GCATCAACAACTTCAATGTC	CCAAGAACTCATCAGAGAATC

a The underlined texts refer to the restriction enzyme sites, BamHI and XhoI respectively, embedded in the PCR primers.

### Pulldown experiments in S. mutans with biotinylated 2B5.

S. mutans was grown to stationary phase in THB, and bacterial cells were then collected by centrifugation and lysed by sonication in NETN buffer (20 mM Tris-HCl, 100 mM NaCl, 0.2% NP-40, 1 mM EDTA, pH 7.0). Biotin or biotinylated 2B5 (0.1 μmol) was added to streptavidin-coated beads (Sigma, USA) and incubated at 4°C for 2 h. Biotin immobilized beads were used as a negative control to exclude any potential nonspecific interaction between biotin and recombinant VicR. The conjugated beads collected by centrifugation and washed three times by NETN buffer were then incubated with the prepared cell lysates with gentle shaking overnight at 4°C. Proteins bound to the biotinylated compound were captured on the beads, which were eluted using elution buffer (20 mM Tris-HCl, 100 mM NaCl, 0.2% NP-40, 1 mM EDTA, pH 2.5). Proteins bound to untreated beads or pretreated beads were used as negative controls. The eluents were evaluated by SDS-PAGE analysis, and proteins were stained by Coomassie brilliant blue. Bands that appeared only in the samples treated with the biotinylated compound were excised and subjected to LC/MS/MS analysis to identify proteins as previously described (63).

### Cellular thermal shift assay.

S. mutans cells cultured in THB broth were harvested and washed with phosphate-buffered saline (PBS). The cell suspensions were freeze-thawed three times in a −80°C freezer and sonicated with the following sequence: 5 s on and 10 s off for 15 min. The soluble fraction (lysates) was separated from the cell debris by centrifugation at 20,000 × *g* for 20 min at 4°C. The prepared cell lysates were diluted with NETN buffer and divided into two aliquots. One aliquot was treated with compound 2B5 and the other aliquot with the vehicle control, 1% DMSO. After 10 to 30 min incubation at room temperature, the respective lysates were divided into smaller aliquots of 50 μL each and heated individually at different temperatures as specified for 3 min using the PCR machine followed by cooling down at room temperature for 3 min. The heated lysates were centrifuged at 20,000 × *g* for 20 min at 4°C in order to separate the soluble fractions from the precipitates. The supernatants representing thermal stable fractions were transferred to new Eppendorf tubes and subjected to SDS-PAGE followed by Western blotting using VicR-specific antibody (46). All buffers were supplemented with complete protease inhibitor cocktail as previously described (62).

### Expression and production of recombinant VicR proteins and other response regulators.

Recombinant proteins were generated as follows: *vicR* and *smu.1547* were amplified from genomic DNA of S. mutans by PCR using specific primer pairs (listed in Table 3). The amplified fragments were restricted with BamHI and XhoI, cloned into the BamHI*/*XhoI site of pET-SUMO vector (64), and transformed into XL1-Blue competence cells (Stratagene, USA). The plasmids extracted from E. coli XL1-Blue were confirmed by sequencing and restriction digestion and then transformed into E. coli BL21(DE3) competent cells to express and produce recombinant VicR and Smu.1547 as previously described (64). Isopropyl-β-d-thiogalactopyranoside (IPTG) (1 mM) was added to bacteria grown to an OD_600_ of 0.5 and incubated at 18°C overnight with shaking to induce protein expression. DNA fragments encoding N-terminal (1 to 125 amino acid residues) and C-terminal VicR (126 to 235 amino acid residues) were PCR amplified using primers listed in Table 3, cloned, and ligated into pET28a to generate VicR-N- and VicR-C-containing plasmids, respectively. The plasmids were confirmed by sequencing and then transformed to E. coli BL21(DE3) to obtain kanamycin-resistant transformants (50 μg/mL). Production of proteins was induced with 0.2 mM IPTG. The induced cells were harvested after 16 h of incubation at 18°C and subjected to a two-step protein purification described below.

### Purification of His-tagged VicR and its N- and C-terminal recombinant proteins.

Harvested cells were subjected to cell lysis by sonication, and the lysates were then loaded onto Ni-nitrilotriacetic acid (Ni-NTA) His-Bind resin slurry column (Novagen, USA). Recombinant proteins bound to the column were eluted and then digested with sumo-protease to cleave the Sumo tag. After dialysis, the cleaved proteins were passed through Ni-NTA His-bind column and collected from flowthrough fractions, which were further purified by gel filtration as previously described (64). The purity and quantity of proteins was established by SDS-PAGE analysis and protein staining.

### Crystallization and structural determination of VicR-N.

Crystals of VicR-N appeared in the condition of 0.1 M HEPES, pH 7.0, 20% PEG 3350, 0.1 M sodium chloride by hanging drop method of vapor diffusion at 20°C. The diffraction data of VicR-N crystals were collected at Argonne National Laboratory APS ID 22 beamline. The resolution of the data reaches 2.3 Å, and the crystal belongs to space group P65 with cell constants a = 53 Å, b = 53 Å, c = 196 Å, corresponding to two molecules per asymmetric unit. Data were processed and scaled with HKL2000 (65). The structure was determined by molecular replacement using its homologous structure (PDB entry number 2A9R) as a search model. The structure determination and refinement were performed in Phenix (66). The final model has an R factor of 0.1994 and an R-free value of 0.2589. Data collection and refinement statistics for VicR-N are shown in Table 1.

### Ligand docking of 2B5 within the VicR-N structure.

Docking 2B5 to VicR-N was carried out with AutoDock 4 (67). The VicR-N was given as a rigid three-dimensional construction, and the 2B5 was allowed for rotations to perform the docking. Data on the position and free energy of the 2B5 docking across the entire VicR-N surface were obtained. The negative and low value of binding (ΔG) indicates strong favorable bonds between VicR-N and 2B5, and 2B5 was in its most favorable conformations. All structure figures were created by PyMol (68).

### Binding kinetics determined by Octet Red system.

Bio-layer interferometry technology (Octet Red system; ForteBio) was employed to measure the binding between VicR and 2B5. Both biotinylated-2B5 and unlabeled 2B5 were used. The biotinylated 2B5 (40 μM) was immobilized on streptavidin biosensors (SA sensor, ForteBio) in PBST buffer (PBS with 0.025% Tween 20). After washing and removing excess, the biosensors were incubated with 2-fold dilutions of VicR (association, 100 to 1.5 μM), and then the biosensors were incubated with the PBST buffer to determine the dissociation rate. The opposite binding order with Ni-NTA biosensor (NTA sensor; ForteBio) was also evaluated using the same method. A total of 40 μM VicR protein was immobilized on NTA sensors in PBST buffer. The unlabeled 2B5 compound was diluted in different concentrations (320 to 5 μM) for the binding. The dissociation rate (*K_d_*) and relative dissociation constant for the binding between 2B5 and VicR were analyzed using Octet Red system data analysis software.

### Effects of 2-AI derivative on expression of *vicR* and other genes.

Compound 2B5 at 5, 10, and 20 μM or 1% DMSO control was added to either S. mutans or S. aureus cultures grown to an early log phase. One hour and 2 h after the addition of the compound, the bacterial absorbance at 470 nm was measured, and bacterial cells were harvested by centrifugation. The same number of cells were lysed, treated, and then subjected to SDS-PAGE analysis as described above. Western blotting was carried out using VicR antiserum (1:5,000 dilution). Streptococcal heat shock protein 70 was used as an internal control to normalize the amounts of proteins used for each sample.

### Pulldown of purified recombinant VicR proteins by biotinylated 2B5.

Streptavidin beads were used to pull down proteins bound to biotinylated 2B5. Streptavidin beads were washed with NETN buffer 3 times and then incubated with the biotinylated compound for 1 h. The prepared beads were washed 3 times with NETN buffer before being incubated with purified recombinant proteins (VicR, VicR-N, and VicR-C) in NETN buffer for at least 1 h. The beads were washed and eluted with elution buffer, and the eluents were subjected to SDS-PAGE and Coomassie blue staining or Western blotting.

### Rat model of dental caries studies.

S. mutans colonization and cariogenicity studies were carried out using a rat model of dental caries as previously described (46, 69, 70). Fischer 344 rats at 20 days of age were randomly assigned into three experimental groups of 6 animals. Rats were then infected with S. mutans UA159 for three consecutive days and provided a caries-promoting Teklad Diet 305 containing 5% sucrose (Harlan Laboratories, Inc., Indianapolis, IN) and sterile drinking water *ad libitum*. One group of rats was then treated with the lead 2-AI compound at 100 μM while the second group was topically treated with a vehicle control twice daily for 4 weeks beginning 10 days postinfection. The third group was treated with 250 ppm fluoride as a positive control in the same manner since fluoride has been used as a golden standard to prevent dental caries (71). Following each treatment, drinking water was withheld for 60 min. Animals were weighed at weaning and at the termination of the experiment. The animals were euthanized, and their mandibles were excised for microbiological analysis of plaque samples on MS agar plates and for scoring of caries by the method of Keyes (72). Differences were compared statistically by analysis of variance (ANOVA).

### Statistical analysis.

Statistical analyses were carried out by the GraphPad Prism software 9.3.1, and all data are denoted as mean ± standard deviation (SD) unless otherwise specified. Differences are considered to be significant with *P* values of <0.05 (*), <0.01 (**), <0.001(***), or <0.0001 (****).

### Ethics statement.

All experimental protocols were approved by University of Alabama at Birmingham Institutional Animal Care and Use Committee (IACUC-09213). The methods were carried out in accordance with the relevant guidelines and regulations.

### Data availability.

Coordinates and structure factors have been deposited in the Protein Data Bank (PDB) under accession number 8FK2.
