# Supplementary material for: Small Molecule Attenuates Bacterial Virulence by Targeting Conserved Response Regulator
Source: mBio. 2023 Apr 19;14(3):e00137-23. doi: 10.1128/mbio.00137-23 (PMC10294662; doi:10.1128/mbio.00137-23)
Supplement: FIG S1 [file mbio.00137-23-s0001.pdf]

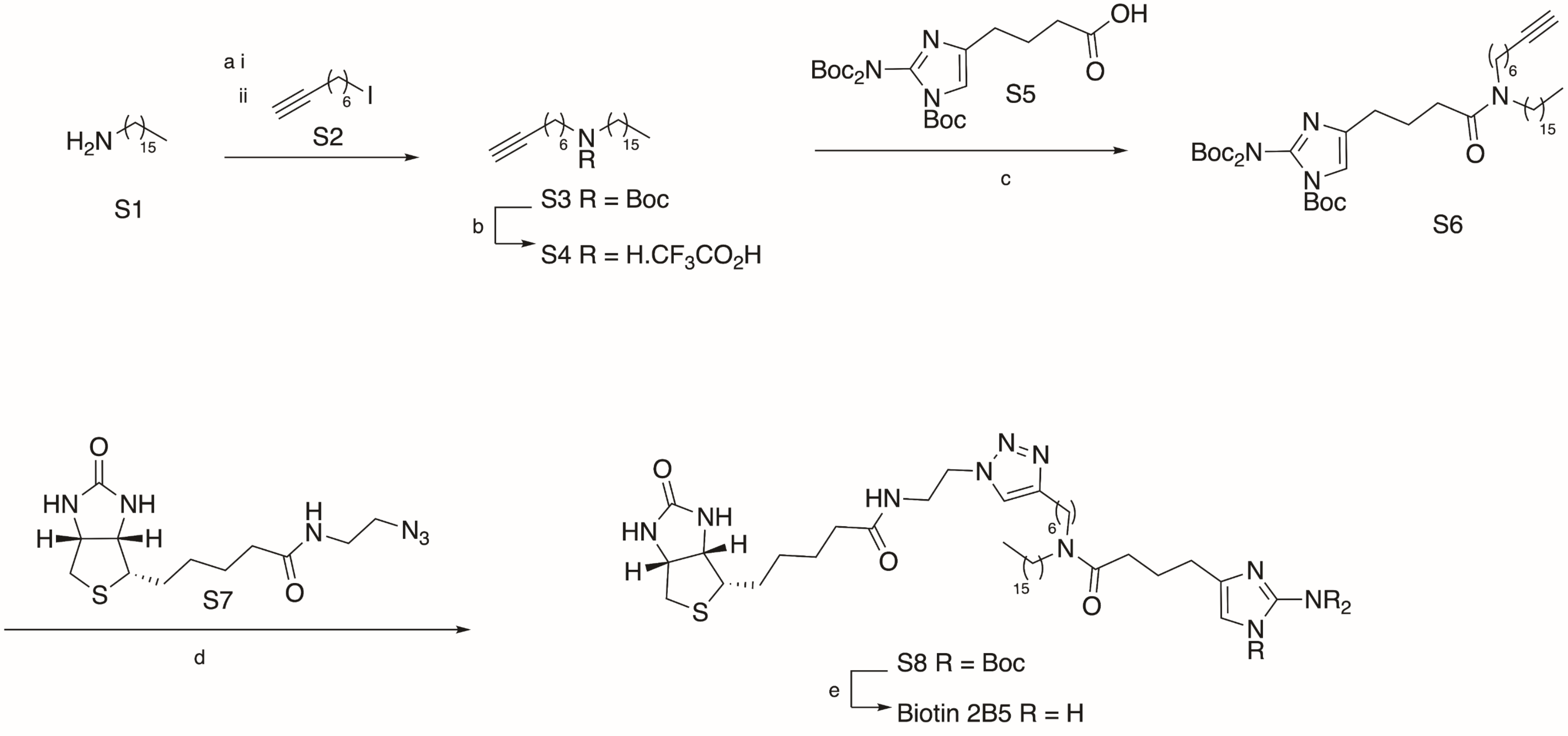


Reagents and conditioins: a) i) $Boc_2O$, DCM, 0 ˚C - rt, 18 h; ii) NaH, DMF, toluene, 0 ˚C - rt, 18 h, 80%; b) TFA, DCM, 0 ˚C - rt, 18 h, 83%; c) EDC, HOBt, DIEA, DMF, rt, 48 h 70%; d) $CuSO_4$, soodium ascorbate, $^{t}BuOH$, $H_2O$, DCM, 65%; e) TFA, DCM, rt, 2 h, then HCl, MeOH 68%.

Supplemental Figure 1. Synthesis of biotinylated 2B5
