# Supplementary material for: Small Molecule Attenuates Bacterial Virulence by Targeting Conserved Response Regulator
Source: mBio. 2023 Apr 19;14(3):e00137-23. doi: 10.1128/mbio.00137-23 (PMC10294662; doi:10.1128/mbio.00137-23)
Supplement: FIG S2 [file mbio.00137-23-s0002.pdf]

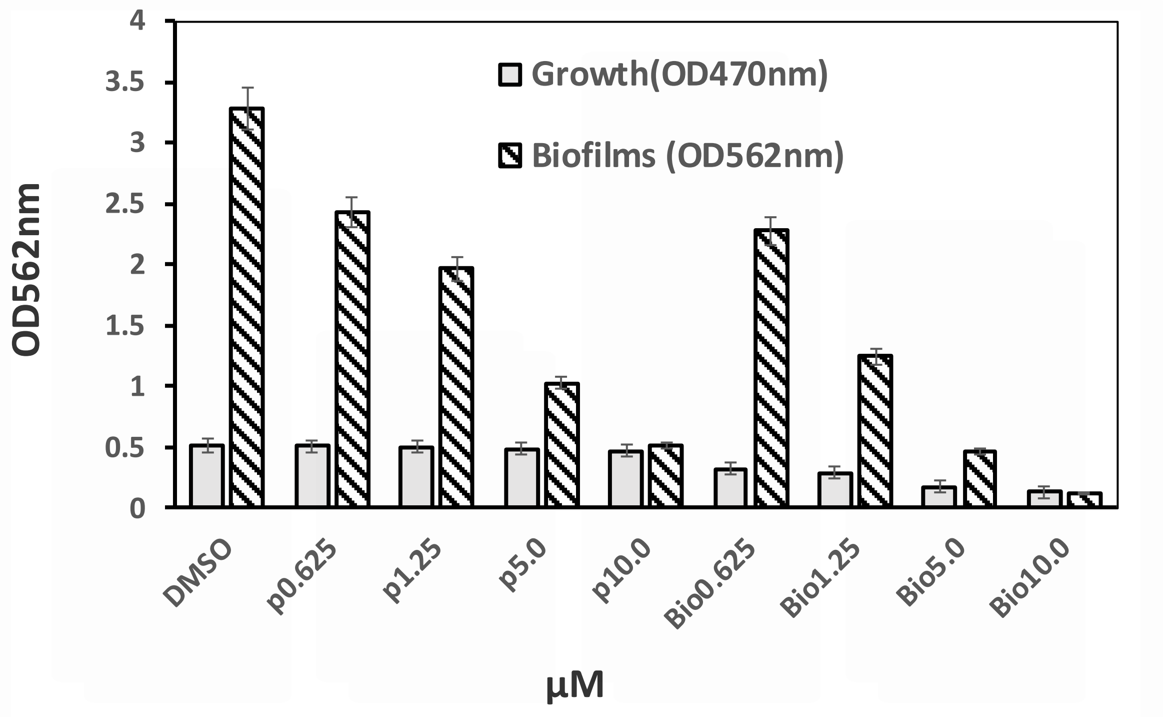

<u>Supplemental Figure 2</u>. Biotinylated 2B5 (Bio) was more potent than its 2B5 precursor (p) in the inhibition of *S. mutans* biofilm formation.
