# Supplementary material for: Small Molecule Attenuates Bacterial Virulence by Targeting Conserved Response Regulator
Source: mBio. 2023 Apr 19;14(3):e00137-23. doi: 10.1128/mbio.00137-23 (PMC10294662; doi:10.1128/mbio.00137-23)
Supplement: FIG S3 [file mbio.00137-23-s0003.pdf]

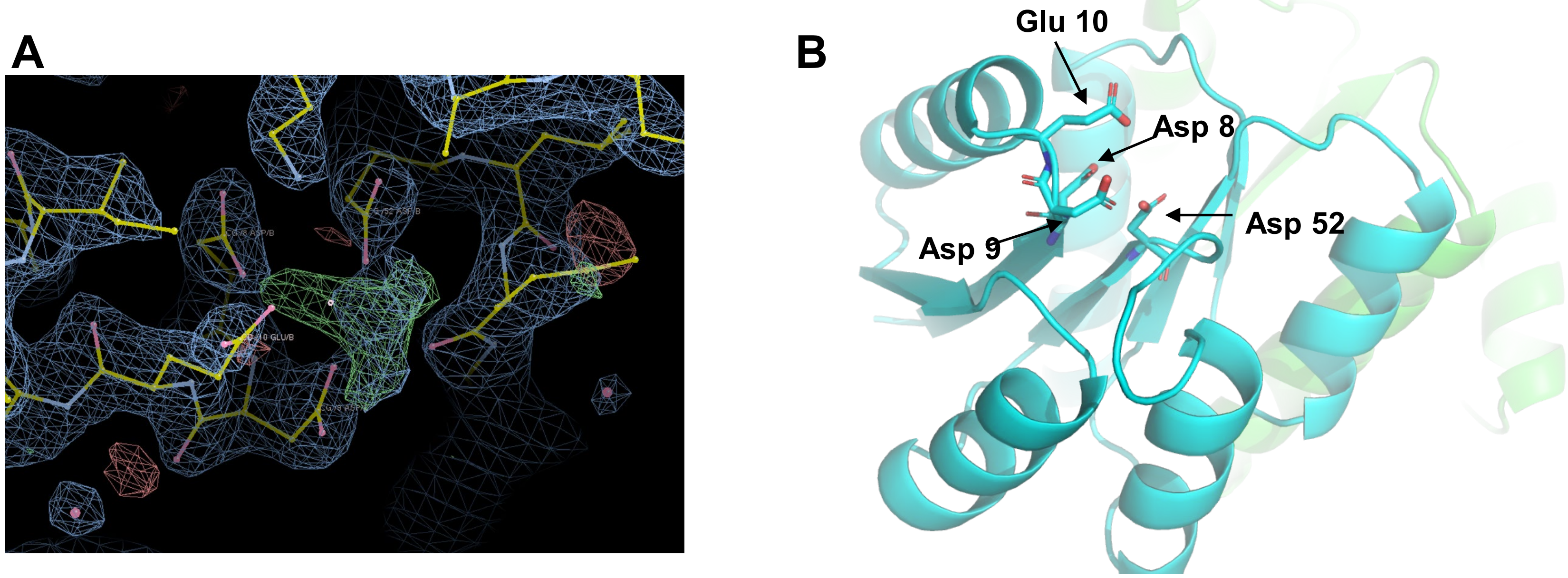

Supplemental Figure 3. The binding pocket from the structural map of VicRN. **A**, a density exists in the experimental structure map that is surrounded by four negatively charged amino acids. **B,** The four negative charged amino acids were labeled with arrows.
